# A Discussion of the Role of Frontoparietal Activity in Cognition

**DOI:** 10.1100/tsw.2007.267

**Published:** 2007-10-22

**Authors:** Nicholas Hon

**Affiliations:** Department of Psychology & Centre for Life Sciences, National University of Singapore, 11 Law Link, 117570, Singapore

**Keywords:** frontal, parietal, attention

## Abstract

A consistently observed pattern in the functional brain imaging literature is that of joint frontal and parietal activation. Because this pattern of activation has been observed under many different experimental conditions and when different cognitive domains have been tested, it is likely that frontoparietal activity plays a very general role in cognition. This article considers one such possible role – the representation of behaviourally relevant information.

Frontal and parietal association cortices have been the subject of intense biobehavioral research. A typical aim of this type of research is to relate frontal and parietal functioning to cognition and behavior. Given the level of interest in these two regions of the brain, it is unsurprising that we now have a reasonably rich picture of the cognitive and behavioral activities that they are involved in. Early investigations utilizing methods like single-cell recordings often examined these two disparate parts of the brain separately[1,2]. Whole brain imaging techniques like positron emission tomography (PET) and functional magnetic resonance imaging (fMRI) allow for another interesting avenue for research. Although these methods lack the fine-grained spatial resolution of others (e.g., invasive cell recordings), they nonetheless give insight into patterns of activation across the whole brain. One such pattern is that of joint frontal and parietal activation, a pattern observed in many different studies. In this review, I discuss frontoparietal activity and its relation to cognition, suggesting that it plays a very general role in cognitive activity.

## INTRODUCING THE FRONTOPARIETAL NETWORK

An inspection of the functional brain imaging literature reveals a ubiquitous pattern: the joint activation of frontal and parietal areas. What is interesting about this pattern is that the locations of the frontal and parietal sites are very similar across studies, even when very different experimental protocols and stimulus sets are utilized[3,4,5]. For example, similar frontoparietal activation has been observed with a task manipulating the amount of semantic retrieval required[6] and one manipulating the amount of planning behavior required[7]. Typically, brain activity is related to the conceptual focus of the study that yields it. Frontoparietal activity has been observed in studies of different cognitive operations and, accordingly, has been interpreted in relation to the conceptual foci of those studies. The result is that many different views have been offered regarding the function of frontoparietal activity. For example, it has been implicated in both working memory[8] and in language processing[9]. In some instances, there have been attempts to ascribe different functions to frontal and parietal components of the network[e.g., 3,8].

The consistency with which this pattern of activation has been observed begs the question of how similar these activations actually are across studies. Duncan and Owen made a systematic study of exactly this issue. They plotted the peaks from a number of different imaging (fMRI and PET) studies[5]. The studies came from five different classes of cognitive demand: response conflict, task novelty, working memory load, working memory delay, and perceptual difficulty. When the peak frontal activations from these studies were compared, it was noticed that the different cognitive demands engaged very similar frontal areas. Common frontal areas activated by these different tasks included anterior cingulate cortex, dorsolateral prefrontal cortex around the inferior frontal sulcus, and the frontal operculum. When posterior activations were considered, these noticeably clustered around the intraparietal sulcus[4]. The findings of those meta-analyses suggest that the frontal and parietal areas activated by different cognitive demands are extremely similar. This prompts an interesting interpretation for joint frontal and parietal activation: It may support a cognitive operation or resource that is common to many different tasks.

What exactly might this general resource or operation be? Because many tasks seem to rely on it and the nature of these tasks can be very different, one reasonable speculation would be that it is likely to be very basic. One strong candidate proffered by the literature relates to attention. In the following section, I consider frontoparietal activity in studies of attention.

## FRONTOPARIETAL ACTIVITY AND ATTENTION

Studies of attention have consistently reported frontoparietal activation. For example, Corbetta found that covertly directing visual attention to different locations engaged the intraparietal sulcus and dorsolateral prefrontal cortex[10]. This study also contrasted covert shifts of attention with overt ones, and found that both activated similar and overlapping areas in the brain, including those in frontal and parietal lobes. Initially, this finding led to the suggestion that frontal and parietal areas are involved in oculomotor intention and not attention specifically. On that view, in a covert attention task, a saccade may be planned, but not executed. However, various subsequent findings have suggested a different interpretation.

Joint frontal and parietal activation is observed when attention is deployed to nonspatial domains and when neither attention, nor the eyes have to be moved to different locations. Attention to time intervals[11] and internal representations[12] have been shown to activate the frontoparietal network. Most relevant to the current discussion is the finding of a positive relationship between attentional demands and frontoparietal activity. Marois and colleagues found greater frontoparietal activity when subjects performed more attentionally demanding tasks compared to less demanding ones[13]. Critically, this finding was obtained even when the stimuli were presented in foveal vision and required no spatial shift of attention.

Because of the ubiquity of frontoparietal activation in studies of attention, the frontoparietal network has been associated with attentional control[14]. Briefly, attentional control refers to the deployment of attention on the basis of a person’s goals, expectations, or knowledge[14,15]. Possibly the strongest support for this proposal comes from studies that have demonstrated sustained frontoparietal activity throughout the period that attention is *volitionally* deployed. Kastner and colleagues found, with a cuing paradigm, that the components of the frontoparietal network exhibit exactly this quality[16]. In that study, the intraparietal sulcus and prefrontal cortex demonstrated a sustained response that was time locked to the presentation of a cue, and which extended through the period that attention was directed to the cued location. Corbetta and colleagues[14,17] also demonstrated this cue-locked, sustained frontoparietal activity when the cue indicated the direction of a subsequent moving target, suggesting that this activity was not specific to spatial attention. Correspondingly, while most studies have focused on visual attention, it has also been established that attention directed to other modalities engages similar areas. For example, Macaluso and Driver found that the same frontal and parietal sites were activated whether subjects directed attention to visual or tactile stimulation[18].

An inspection of the wider literature reveals much the same picture: Tasks that require attentional control activate frontal and parietal areas. In the Duncan and Owen meta-analysis discussed previously[5], although considering different cognitive demands, the studies included in the meta-analysis all involved contrasts pitting versions of tasks requiring more attentional control with those requiring less. It should be noted that attentional control can be required by different aspects of a task and engaged during different phases of task performance. Attentional control can, for example, be required during the preparation stage prior to the presentation of stimuli. Correspondingly, when a pretarget cue indicates the requirement of volitionally controlled shifts of attention between task or cognitive sets, greater frontoparietal activation is observed relative to when the cue indicates that no such shift is required[19,20]. And obviously, attentional control can be required during performance of a task. This is particularly so when tasks require the resolution of conflict or on-line deliberate selection of information from a number of alternatives. Consistent with this, Thompson-Schill and colleagues found that versions of tasks that require more controlled selection (from alternative responses) evoke greater frontal and parietal activation than versions requiring less such selection[21]. Similarly, patients with frontal lobe damage have been known to have difficulties with keeping irrelevant information “out of mind”[22]. The proposal that the frontoparietal network is associated with attentional control is also buttressed by the finding that, as attentional control becomes less important for task performance (e.g., with increased practice or automaticity), frontoparietal activity diminishes. For example, Petersen and colleagues found that, as a maze tracing task became more automatic, prefrontal and parietal cortex were recruited less and less[23].

## FRONTOPARIETAL ACTIVITY AND THE REPRESENTATION OF RELEVANT INFORMATION

But, what role is played by the frontoparietal network in attentional control? A typical view is that the frontoparietal cortex holds descriptions of behaviorally relevant information[1,24,25,26]. This higher-level representation then guides, via feedback connections, processing in other brain systems such that overall processing converges on what is relevant for behavior. On this view, attentional control is said to be exerted as these higher-level representations influence related processing. Electrophysiological studies have demonstrated emphatically that processing in primary and secondary brain systems are influenced by task or behavioral relevance. For example, the responses of early and late visual cells are determined by task-relevant stimuli, even when irrelevant stimuli are presented in the same receptive field, and when the cell is less sensitive to the relevant stimulus than the irrelevant one[27,28]. Such relevance-related effects are observed after the initial on-discharge of the cells, suggesting that they may be the result of influences from other brain systems. Plausibly, these relevance effects originate from frontoparietal representations coding for task-relevant information.

Frontal and parietal cortices appear to have the requisite properties needed to perform this guiding function. First, they have extensive connections to each other and many other brain regions[29,30,31,32], which would allow them to influence processing in other brain systems. Deactivating the prefrontal cortex by cooling, for example, can modulate visual responses to a behaviorally relevant stimulus[33]. Second, cells in both these areas are able to code for abstract information relating to task performance. Frontal cells, for example, have been observed to code for stimulus categorizations[34], task rules[35], and even expected rewards[36]. In addition, cells have been found in frontal and parietal cortex that are able to flexibly tune their response sensitivities to coincide with changing task demands, suggesting a mechanism by which frontal and parietal cortex might play a role in many different cognitive demands. For example, Freedman et al. found, in a monkey trained to make cat-dog discriminations, frontal cells that distinguished between cats and dogs[37]. When this same monkey was retrained to perform discriminations between cat-dog pairs, a proportion of the originally tested frontal cells now distinguished between the different pairs, abandoning their original coding preferences. Parietal cells are also known to be able to flexibly code for task-relevant information: Location-sensitive parietal cells demonstrated sensitivity to color when color was task relevant[38]. Finally, because tasks are often performed over a period of time, it is important that representations of task-relevant information be maintained for some duration. Frontal and parietal cells appear to have this ability. For example, frontal cells coding for task-relevant information have been observed to maintain their activity over time[35], with this sustained activity being resistant to the presentation of task-irrelevant distractors, even when these are attended and processed[39].

## FRONTOPARIETAL ACTIVITY EVEN WITH PASSIVE RECEPTION OF INFORMATION

The preceding discussion revolved around the idea that a frontoparietal network supports attentional control by housing descriptions of relevant information. Attentional control is said to be exercised when these higher-level representations influence processing in other brain systems such that overall processing is related to what is relevant to the organism

An interesting question pertains to what exactly should be considered relevant information. In a typical experiment, relevance is easily manipulated and conveyed to a subject. For example, with humans, one merely needs to indicate by verbal or written instructions that something is a target for some behavior or not. With animals, one can indicate relevance by pairing a stimulus with a reward. In a typical experiment, then, an experimenter assesses whether this information has been successfully coded or represented by observing whether a subject makes the appropriate responses to the specified targets. A critical point, therefore, is that relevance is often confounded with the requirement of some task performance. As such, with experiments that utilize active tasks, it is possible that any observed frontoparietal activity reflects general task-related processes like stimulus-response mapping and/or decision processes.

A strong test of the proposal that the frontoparietal network acts to represent relevant information per se would be to observe whether or not it is engaged by relevant information even when task demands are minimized or absent. In such a scenario, relevant information would be represented, but would not be used for task-related processing. My colleagues and I studied this issue recently. In our study, subjects simply paid attention to one of two overlapping streams of equivalent visual events[40]. The subjects were neither required to perform a task, nor make any decisions or responses. In addition, they were also never required to shift attention. The idea behind this experiment is that information that is attended would be deemed "relevant" by the mind/brain. When we pay attention to something, it becomes the focus of our mental activities, essentially conveying it higher status or priority than things not attended to. Therefore, what is attended is relevant, whether or not we have to do anything with this information. Consistent with this, pronounced frontoparietal activity was observed in association with attended visual events, even when these events required no further task-based processing. Unattended visual events did not produce any such activation. We also observed that this frontoparietal activity from passive reception of attended information was not stimulus specific. Primate studies have, on occasion, reported frontal and parietal activity with passive "tasks"; however, those tended to use stimuli that were ecologically salient[e.g., 41,42]. In our study, frontoparietal activity was observed even when the attended stimuli were largely meaningless (i.e., novel "nonsense" line drawings), suggesting that this pattern of activity is not determined by the ecological importance of the stimuli being perceived.

Although not their main aim, other studies have reported results that are compatible with the idea that frontal and parietal cortex act to represent what is relevant. Recently, a number of studies have reported frontoparietal activity in relation to changes to (or updates of) the contents of awareness (i.e., what we are aware of at any moment in time). For example, shifts in the percept experienced when two images are presented under conditions of binocular rivalry are accompanied by dorsolateral prefrontal and intraparietal activity[43,44]. Similar activations have been reported in relation to shifts in the conscious

"interpretation" of bistable figures[45] as well as when subjects become aware of patterns in sequences of stimuli[46]. Possibly, as an image or idea gains dominance over the percept, it becomes the focus of attention and therefore is considered relevant.

Additionally, it has also been found that novel and distinct intrusions in an otherwise predictable sequence of events evoke activity in the prefrontal cortex and intraparietal sulcus[47]. It is plausible that the distinctiveness or salience of a stimulus can "capture" an observer's attention, making the distinct stimulus momentarily relevant.

## FUTURE DIRECTIONS

This article had, as its aim, a brief discussion of a possible role for joint frontal and parietal activity in cognition, a pattern consistently observed in the brain imaging literature. The fact that the frontoparietal network has been observed in so many different experimental settings suggests that it acts to support a very general cognitive function. Taken together, the literature suggests that the frontoparietal network may support attentional control by representing information that is relevant to the organism. Cognitive or attentional control, then, is exerted when frontoparietal representations guide or bias processing in other parts of the brain such that overall processing converges on what is relevant to the organism.

While some headway has been made in determining the role of this network in cognition, many other issues remain to be examined. In this concluding paragraph, I highlight two issues that would benefit from further investigation. The first issue pertains to the exact contributions of frontal and parietal areas to the representation of relevant information. One possibility is that frontal and parietal neurons perform exactly the same function when representing relevant information[e.g., 48]. Another possibility is that frontal and parietal areas play different roles. Lesion studies typically report that frontal and parietal patients suffer different symptoms[22,49], suggesting the possibility that the frontal and parietal cortex may contribute differently to the overall function of the network. Subsequent research might clarify this issue. A second issue that would benefit from greater inquiry relates to our understanding of the functioning of the network as a whole. A recent study showed that the network as a whole can behave differently depending on how relevance is determined[50]. As noted earlier, relevance can be indicated by verbal instructions or association with rewards. However, an object can also become relevant based on how physically distinctive or salient it is. Buschman and Miller found that, even though the frontoparietal network as a whole responded to both types of relevant information, the activity of frontal and parietal cells synchronized at different frequencies depending on how relevance was defined[50].

## ACKNOWLEDGMENTS

This work was supported by grant R581000057112/ 133 from the National University of Singapore.
